# Clinical Utility of Amino Acid PET-MRI in Children with CNS Neoplasms: A Territory-Wide Study from Hong Kong

**DOI:** 10.3390/cancers17193233

**Published:** 2025-10-04

**Authors:** Evelyn R. Lu, Pui Wai Cheng, Sherman S. M. Lo, Chloe W. Y. Siu, Eric C. H. Fu, Jeffrey P. W. Yau, Anselm C. W. Lee, Kwok Chun Wong, Elaine Y. L. Kan, Sarah S. N. Lau, Wilson W. S. Ho, Kevin K. F. Cheng, Emily K. Y. Chan, Ho Keung Ng, Amanda N. C. Kan, Godfrey C. F. Chan, Dennis T. L. Ku, Matthew M. K. Shing, Anthony P. Y. Liu, Deyond Y. W. Siu

**Affiliations:** 1 Department of Paediatrics and Adolescent Medicine, Hong Kong Children’s Hospital, Hong Kongericfu_1668@yahoo.com.hk (E.C.H.F.);; 2 Department of Paediatrics and Adolescent Medicine, School of Clinical Medicine, LKS Faculty of Medicine, The University of Hong Kong, Hong Kong; 3 Scanning Department, St. Teresa’s Hospital, Hong Kong; 4 Department of Radiology, Hong Kong Children’s Hospital, Hong Kong; 5 Department of Neurosurgery, Hong Kong Children’s Hospital, Hong Kong; 6 Department of Neurosurgery, Prince of Wales Hospital, Hong Kong; 7 Department of Anatomical and Cellular Pathology, Chinese University of Hong Kong, Hong Kong; hkng@cuhk.edu.hk; 8 Department of Pathology, Hong Kong Children’s Hospital, Hong Kong; 9 Neuro-Oncology Section, Hospital for Sick Children, Toronto, ON M5G 1X8, Canada

**Keywords:** amino acid PET, PET-MRI, central nervous system neoplasms, paediatric brain tumors, germ cell tumor

## Abstract

**Simple Summary:**

This study highlights the value of amino acid positron emission tomography–magnetic resonance imaging (PET-MRI) in improving the diagnosis and management of brain tumours in children. Unlike standard MRI, which lacks information on metabolic activities, this advanced imaging technique helps distinguish high-grade brain tumours from low-grade lesions or non-malignant lesions. Measurable cut-off points to differentiate between the two types of processes were obtained, which can aid future research in tumour classification. The study also showed that amino acid PET-MRI influenced treatment decisions in 69% of cases in a territory-wide referral centre, suggesting its strong clinical relevance. For the research community, this work supports the broader use of amino acid PET-MRI in paediatric neuro-oncology and encourages further studies to refine non-invasive diagnostic tools and improve personalized treatment planning. It sets the stage for prospective, multicentre studies and contributes valuable data to the growing field of hybrid imaging in childhood brain tumours.

**Abstract:**

**Background**: Amino acid tracer positron emission tomography–magnetic resonance imaging (PET-MRI) was shown to be superior to MRI alone for evaluating central nervous system (CNS) tumours in adults. This study aimed to investigate the utility of amino acid PET-MRI in children with CNS tumours. **Methods**: We reviewed the amino acid PET-MRI findings of children with suspected or confirmed CNS neoplasms managed in a territory-wide referral centre in Hong Kong from 2022 to 2025. Maximal standardized uptake values (SUVmax) were captured, and tumour-to-background SUVmax ratios (TBRmax) were measured with reference to adjacent or contralateral normal brain structures. Comparisons were made among patients with clinical high-grade and low-grade/non-neoplastic lesions. **Results**: Thirty-seven patients were included, with 63 PET-MRIs performed. PET-MRI was performed as part of initial diagnostics in 41% of the cases, for response assessment in 48%, and evaluation of residual/relapsed disease in 11%. High-grade lesions had a significantly higher SUVmax and TBRmax compared to low-grade/non-malignant lesions (median SUVmax 3.7 vs. 1.6, *p* = 0.00006; median TBRmax 2.06 vs. 0.91, *p* = 0.00002). Optimal SUVmax and TBRmax cut-offs by ROC analysis were 2.38 and 1.62, respectively. Similar performance was reproduced by focusing on the subset of patients with suspected CNS germ cell tumours (CNS-GCT). The impact of amino acid PET availability is considerable, as clinical management was modified in 65% of patients. **Conclusions**: Our study demonstrates the performance and clinical utility of amino acid PET-MRI in the management of children with CNS pathologies. Amino acid PET-MRI contributes to the diagnosis, monitoring, and treatment guidance of these patients, providing crucial information for decision-making.

## 1. Introduction

Paediatric brain tumours comprise a heterogeneous disease spectrum, often presenting significant challenges in diagnosis and treatment response monitoring. The deep-seated locations frequently restrict the feasibility of invasive procedures, while the absence of reliable biomarkers for disease monitoring complicates management. Additionally, the increasing complexity of tumour classification necessitates more advanced approaches in the management of paediatric brain tumours, such as targeted therapy, immunotherapy, and radiotherapy, which could also complicate radiological interpretations.

Magnetic resonance imaging (MRI) is the standard imaging modality in neuro-oncology due to its high resolution for anatomical details. The use and standardization of relevant sequences, such as those recommended by the Response Assessment in Paediatric Neuro-Oncology working group (RAPNO), allows objective assessment of treatment response [1]. However, anatomical scans alone may be inconclusive in certain situations, such as evaluating non-enhancing gliomas, differentiating between treatment-related changes and active disease, and distinguishing radionecrosis and pseudoprogression from true progression. Amino acid tracer positron emission tomography (PET) is an established, novel imaging modality in adult neuro-oncology. Consensus guidelines have been published recently for its use in adult gliomas, with indications such as differentiation of high-grade tumours, prognostication, definition of optimal biopsy sites, delineation of tumour extent, differentiation of recurrence from treatment-induced changes, and response assessment [2,3,4,5,6,7,8,9]. Unlike F18-fluorodeoxyglucose (F18-FDG) PET, where high physiological background brain uptake results in poor tumour-to-background ratio, amino acid tracers are selectively taken up by various brain tumours.

Two types of tracers that are commonly used for amino acid PET images are C11-methionine (MET) and O-(2-[18F] fluoroethyl)-l-tyrosine (FET). Amino acids play an essential role in many crucial cellular processes, with cellular uptake by both passive diffusion and governed by transporters such as L-type amino acid transporters (LATs) and alanine-serine-cysteine transporters (ASCT). Most gliomas significantly overexpress LAT1 and ASCT2, whilst normal brain tissue has low expression of these transporters, resulting in high tumour-to-normal tissue contrast in amino acid PET. Unlike gadolinium contrast, used in MRI, the uptake of amino acid radiotracers into actively proliferating neoplasms is not limited by an intact blood–brain barrier. For MET, the main metabolic pathway after being transported into tumour tissue is protein incorporation, but it can also be metabolized via smaller pathways [10,11]. The major limitation of MET PET is the short half-life of the C11-radiotracer (20 min), making an on-site cyclotron facility a prerequisite. Another amino acid tracer, FET, has similar uptake affinity and image contrast compared with MET. For FET, there is little metabolism after being transported into tumour tissue, and it can accumulate in the tumour tissue largely unchanged [10,11]. FET is metabolically inert, allowing for the study of uptake kinetic analysis to provide dynamic assessments that distinguish high-grade from low-grade gliomas. FET has a long half-life of 110 min, making it easier to produce and handle, and it can perform dynamic assessments. The pairing of amino acid PET with MRI enables the evaluation of both tumour morphology and metabolic activity in a single visit, resulting in a more precise assessment of tumour grade and extent, as well as contributing to prognostication and treatment response monitoring [12,13,14,15,16,17,18,19]. A meta-analysis has also compared different types of tracers used in PET for high-grade gliomas and shown that FET and MET have a comparably higher sensitivity than FDG in differentiating between tumour progression and treatment-related changes [20]. FET has lower uptake in inflammatory cells compared to FDG and MET, making it more specific for differentiating tumoural tissue from inflammation [10,11]. A recent retrospective study combined the amino acid tracers MET and FET in an analysis of the prognostic significance of amino acid PET in adult glioma patients [21]. Several studies have either evaluated the integration of amino acid PET with conventional MRI or focused exclusively on patients with gliomas [22,23,24,25,26,27]. However, its application in paediatric populations is limited, and its role in paediatric clinical management is still largely under research.

Our study aimed to assess the utility of amino acid PET-MRI within a paediatric neuro-oncology cohort from the only referral centre for paediatric cancers in Hong Kong, highlighting the benefits of this imaging modality for diagnosing and monitoring paediatric brain tumours, including central nervous system germ cell tumours (CNS-GCTs). CNS-GCTs are relatively prevalent in the East Asian population and represent approximately 20% of paediatric brain tumours in Hong Kong. Histological diagnosis is achievable if the lesion is accessible for biopsy. Yet the risk for accessing these tumours, which typically arise from the pituitary, pineal, and basal ganglial regions, is considerable. However, delayed diagnoses, leading to disease progression and irreversible morbidities, are frequently observed, primarily due to nonspecific imaging findings and the inherent hazards associated with neurosurgical sampling. Most studies and reviews have focused on the role of amino acid tracer PET in paediatric gliomas, while only a few case reports and series have addressed its utility in CNS-GCT [28,29,30]. Notably, most of these studies employed amino acid PET-CT rather than PET-MRI, rendering our investigation into amino acid PET-MRI among the few to address its utility in the paediatric population (Table 1).

## 2. Materials and Methods

### 2.1. Study Population

This is a retrospective review of all paediatric patients with suspected or confirmed CNS neoplasms who underwent amino acid PET-MRI from January 2022 to June 2025 in Hong Kong. Since 2019, all patients <18 years of age with cancer diagnoses have been treated in the sole territory-wide paediatric oncology centre in Hong Kong, while PET-MRI scans were performed at a private facility pioneering in neural PET services locally. Patient records were retrieved from the Clinical Management System (CMS) in Hospital Authority computers and the Electronic Health Record Sharing system (eHealth), which is an electronic medical record system shared between public and private hospitals in Hong Kong. Diagnosis of the CNS lesion was eventually made by either biopsy or a combination of clinical and radiological findings, per standard clinical approaches. The decision to arrange PET-MRI was made by paediatric oncologists or neurosurgeons in joint clinics or multidisciplinary meetings after reviewing each individual’s case. The most common indications for PET-MRIs included confirmation or refinement of diagnosis, treatment and disease monitoring, and evaluation for residual or relapsed disease when conventional MRI findings were inconclusive. In our cohort, CNS neoplasms were stratified into high-grade and low-grade categories based on histopathological evaluation according to the World Health Organization Classification of Tumours, supplemented by molecular or genetic profiling when available. For low-grade lesions lacking histopathological confirmation, such as thickened pituitary stalk lesions, which pose diagnostic ambiguity, longitudinal clinical monitoring and serial MRI surveillance over a minimum observation period of one year was performed to ensure diagnostic stability and exclude progression.

### 2.2. Imaging Methods and Analysis

In the majority of cases within our cohort, initial diagnostic evaluation adhered to standard clinical practice with conventional MRI. PET-MRI was selectively employed as an adjunctive modality in instances where anatomical MRI findings proved equivocal. For the remaining patients, radiological interpretation integrated PET and MRI datasets concurrently to optimize diagnostic accuracy. The two types of tracers used for PET-MRI were MET and FET. PET-MRI scans performed prior to May 2023 employed C11-MET, while those conducted after May 2023 utilized F18-FET due to its longer half-life and thus greater ease of handling. Both tracers were generated by an on-site cyclotron within the hospital. The height and body weight of the patients were documented before the scan for measurement of the standardized uptake values (SUVs). Before scanning, patients were asked to empty their bladder for maximum comfort during the study and to reduce the absorbed dose to the bladder. If sedation was required, it was to start 20–60 min before the examination. After 4 h of fasting, PET images were acquired on a Biograph mMR PET/MRI 3.0-T scanner (Siemens Healthcare, Erlangen, Germany) 20 min after intravenous injection of amino acid tracers (either MET or FET), with a single-bed-position PET acquisition of 20 min of the brain obtained. Standard 4-tissue (air, lung, water, and fat) MRI Dixon-based attenuation maps were acquired simultaneously with an additional bone map of the head for attenuation correction. Multiplanar PET images were reconstructed using the manufacturer’s standard algorithm (using Syngo.via, MM oncology protocol, Siemens Healthcare).

Simultaneously obtained MRI series were acquired according to the standard scanning protocol, which includes axial and 3D T1-weighted, T2-weighted, fluid-attenuated inversion recovery (FLAIR), diffusion-weighted imaging (DWI), susceptibility-weighted imaging (SWI), and 3D gadolinium-enhanced T1-weighted imaging. Advanced MRI techniques, such as MR spectroscopy (MRS), perfusion imaging, and diffusion tensor imaging (DTI) were incorporated as indicated. The 3D T1-weighted sequence was used for attenuation correction and image fusion with the PET data.

Both qualitative and semi-quantitative evaluations were performed on each PET-MRI scan. Images were visually assessed by expert neuroradiologists with more than 15 years of experience using standard clinical software (Syngo.via, MM oncology protocol, Siemens Healthcare). Focal uptake visually exceeding local background activity (i.e., surrounding supposed healthy brain parenchyma), with/without MRI-identified morphologic lesions, was targeted for semiquantitative analyses. The maximum standardized uptake value (SUVmax) of each lesion was measured by deploying a spherical region of interest (ROI) to the lesion. The mean physiological brain activity uptake in the healthy appearing cortex of the hemisphere contralateral to the tumour, including grey and white matter, is measured from a large crescent-shaped background volume of interest (VOI) [3]. The maximum tumour-to-background ratio (TBRmax) was calculated with reference to the SUVmax of the background VOI in the contralateral normal cerebral hemisphere. All of the post-treatment surveillance scans were performed on the same scanner, using the same imaging protocol and measuring the SUVmax of all pre-existing lesions and any new lesions to improve the reliability of longitudinal assessment.

The primary outcome of our study was to compare the values of SUVmax and TBRmax between high-grade CNS lesions and low-grade or non-oncological lesions, as defined by histology or current clinical gold standards. Secondary outcomes included subgroup comparisons of SUVmax and TBRmax between CNS-GCT and the corresponding low-grade or non-oncological differential diagnoses. The optimal cut-off values of SUVmax and TBRmax for such delineation were also determined.

### 2.3. Statistical Analysis

Statistical analyses were conducted using RStudio (version 4.4.3). Descriptive statistics for patient characteristics, as well as measurements of SUVmax and TBRmax, were illustrated in terms of mean ± standard deviation, median, and range. Nonparametric comparisons of SUVmax and TBRmax between high-grade and low-grade/non-oncological lesions, as well as between CNS-GCT and low-grade/non-oncological conditions, were performed using the Mann–Whitney U-test, with significance (α = 0.05). Effect sizes were quantified using rank-biserial correlation. Receiver operating characteristic (ROC) curve analysis was applied to identify optimal cut-off values for discriminating high-grade from low-grade lesions, with area under the curve (AUC) and Youden’s index used to maximize sensitivity and specificity. Repeated measures correlation was used to assess the correlation between SUVmax, TBRmax, and CNS-GCT tumour markers such as alpha-fetoprotein (AFP), and human chorionic gonadotropin (HCG), in both serum and cerebrospinal fluid (CSF). Post hoc power analysis was used to estimate the achieved power of the study given the observed effect sizes, sample sizes, and alpha = 0.05.

## 3. Results

### 3.1. Patient Demographics and Clinical Characteristics

Thirty-seven paediatric patients with suspected or confirmed CNS lesions were included in this cohort (twenty-two males and fifteen females; median age, 11.2 years; range, 0.6–17 years at first scan; follow-up period from first scan: median, 21 months, range 0–63 months). Sixty-three PET-MRI scans were obtained, employing the FET tracer in forty-eight scans and the MET tracer in fifteen. Histological diagnoses were obtained in 62% of cases, while the remainder were diagnosed based on clinical and radiological features. Most cases without histological diagnoses were either CNS-GCTs or thickened pituitary stalks. CNS-GCTs can be diagnosed by typical radiological features with or without elevated tumour markers. Twenty-one (56%) patients had an eventual diagnosis of high-grade CNS lesions, including fifteen (40%) with CNS-GCTs, four with high-grade gliomas, and two with medulloblastomas. Eight had low-grade neoplasms (22%), including pilocytic astrocytomas, ganglioglioma, congenital brainstem glioma, and Langerhans cell histiocytosis (one confirmed histologically, the other radiologically). The case of brainstem glioma was not biopsied due to procedural risk; it showed a low-grade lesion on the CSF molecular study and a stable lesion for at least 2 years on serial MRI scans. Non-oncological lesions (n = 8, 22%) included thickened pituitary stalks and demyelinating disease (diagnosed by positive anti-myelin oligodendrocyte glycoprotein antibodies). All cases of thickened pituitary stalk had been followed up for at least 1 year and had demonstrated stable lesions on serial MRI scans. Anatomical sites of interest were the pituitary/suprasellar region (49%), pineal gland (16%), cerebellum (14%), basal ganglia (11%), brainstem (8%), ventricles (8%), and others, such as the frontal lobe, cerebellopontine angle, and spine (Table 2).

Among the 15 patients with CNS-GCT included in this cohort (12 males and 3 females; median age at scan, 14 years; range 8–17) (Table 3), 14 patients were diagnosed with germinoma, while 1 patient had a non-germinomatous germ cell tumour (NGGCT). Five patients had bifocal germinomas, and three patients had disseminated disease at diagnosis (two germinomas, one NGGCT). Nine patients achieved complete remission, five patients were still undergoing active treatment, and 1 patient relapsed. The relapsed case was a disseminated bifocal germinoma that relapsed after 18 months and had achieved disease remission after high-dose chemotherapy with autologous stem cell transplantation (No. 1).

### 3.2. PET-MRI Findings

**Figure 1:**
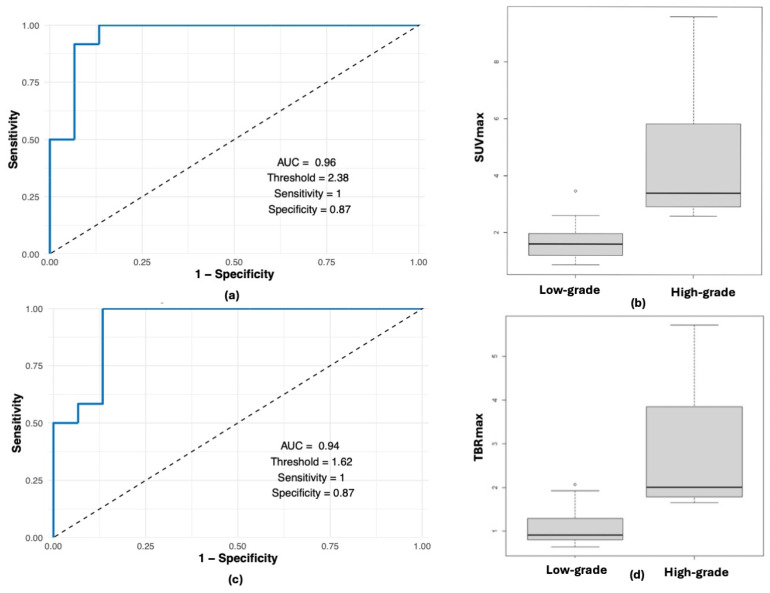
ROC analysis of optimal cut-off values of FET PET-MRI SUVmax and TBRmax to distinguish high-grade CNS lesions from low-grade or non-oncological lesions: (**a**) ROC curve of SUVmax; (**b**) box-plot of SUVmax; (**c**) ROC curve of TBRmax; (**d**) box-plot of TBRmax.

PET-MRI scans were conducted for diagnosis and tumour grading (41%), for treatment and disease monitoring (48%), and for evaluating residual or relapsed disease (11%) (Table 2). Comparisons of SUVmax and TBRmax of all cases in our cohort between MET and FET as PET-MRI tracers, respectively, were made, and no significant differences were found (*p* = 0.26, effect size 0.22; *p* = 0.21, effect size 0.25). Among patients with high-grade lesions evaluated upfront and pre-treatment (n = 12), median SUVmax and TBRmax were 3.7 (range: 2.58–9.6) and 2.06 (range: 1.65–5.72); these values were significantly higher than measurements from upfront images in patients with eventual non-oncological or low-grade lesions (n = 15) (SUVmax median of 1.6, range 0.87–3.47, *p* = 0.00006, power 99%, TBRmax median of 0.91, range 0.64–2.07, *p* = 0.00002, power 99%). Using ROC analysis, the optimal cut-off values of SUVmax and TBRmax to distinguish high-grade CNS lesions from low-grade/non-oncological lesions are 2.38 and 1.62, respectively, with a sensitivity of 100% (CI 92–100%) and specificity of 87% (CI: 73–100%) for both SUVmax and TBRmax (area under the curve 0.96 (95% CI: 0.89,1), 0.94 (95% CI: 0.85,1)) (Figure 1).

### 3.3. CNS-GCT

**Figure 2:**
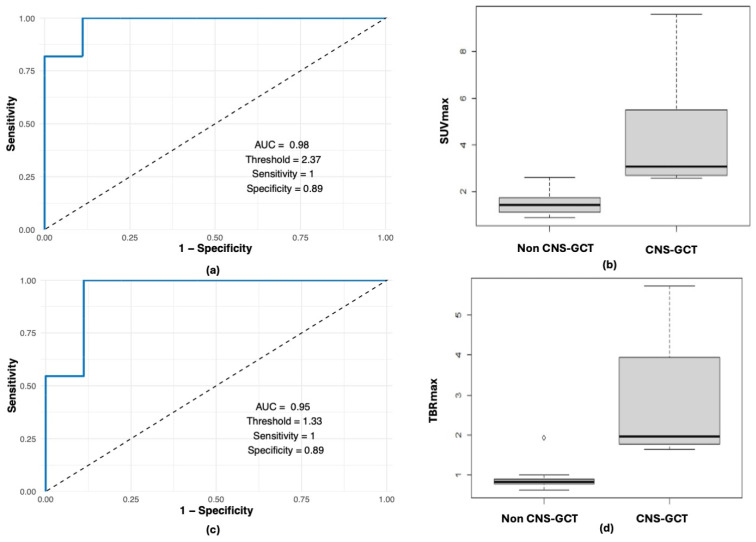
ROC analysis of optimal cut-off values of SUVmax and TBRmax to distinguish CNS-GCT from benign/low-grade non-CNS-GCT: (**a**) ROC curve of SUVmax; (**b**) box-plot of SUVmax; (**c**) ROC curve of TBRmax; (**d**) box-plot of TBRmax.

For the subset of patients with subsequently confirmed CNS-GCTs evaluated upfront (n = 11), median SUVmax and TBRmax were 3.14 (range: 2.56–9.6) and 2.01 (range: 1.65–5.72); these values were significantly higher than measurements from patients with thickened pituitary stalks or low-grade lesions in the pituitary or basal ganglia (n = 9), where CNS-GCT was a differential consideration (SUVmax median of 1.42, range 0.87–2.6, *p* = 0.00005, power 99%, TBRmax median of 0.83, range 0.64–1.93, *p* = 0.0002, power 99%). Using ROC analysis, the optimal cut-off values of SUVmax and TBRmax to distinguish CNS-GCT from benign or low-grade non-CNS-GCT lesions were 2.37 and 1.33, respectively, with a sensitivity of 100% (CI: 82–100%) and specificity of 89% (CI: 78–100%) for both SUVmax and TBRmax (area under the curve 0.98 (95%CI 0.93,1), 0.95 (95% CI, 0.85–1)) (Figure 2).

**Figure 3:**
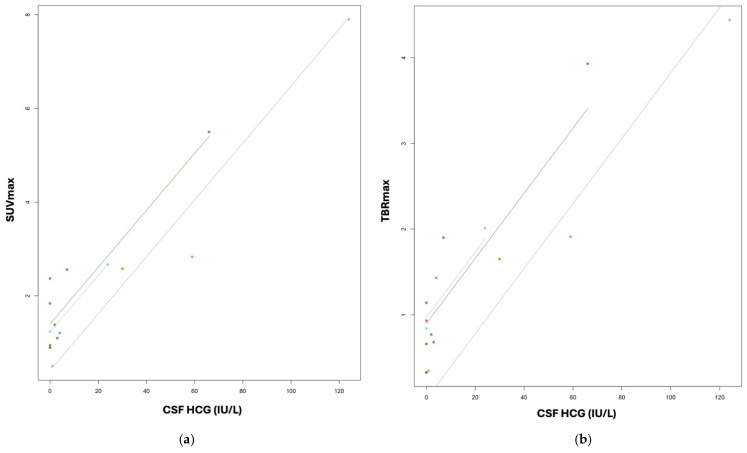
Scatter plots showing the linear correlation between SUVmax and CSF HCG (**a**) and TBRmax and CSF HCG (**b**). The color dots represent each single measurement and the straight lines illustrate the positive linear correlation.

All germinoma patients with elevated tumour markers at diagnosis were followed up for their serial tumour markers at each time point of PET-MRI assessment. A positive linear correlation was found for SUVmax and TBRmax with tumour markers for CNS-GCTs using the repeated-measures correlation. SUVmax and TBRmax were both positively correlated with CSF HCG level (*p* = 0.0004, correlation coefficient 0.95; *p* = 0.003, correlation coefficient 0.96) (Figure 3).

### 3.4. Clinical Impact and Illustrative Cases

We have demonstrated how PET-MRI could provide significant diagnostic information for CNS neoplasms; nonetheless, it is also a valuable tool in guiding decisions and monitoring treatment responses. Clinical impact was modified in 65% of our cases, as shown in Table 4. Specifically, PET-MRI facilitated decision for radiographic surveillance and avoided unnecessary biopsy and resection in 14 patients, and it refined tumour grading or diagnosis in 5 patients with discrepant clinical and histologic features. Treatment decisions were modified in five patients; three underwent surgical resection after PET-MRI indicated high-grade malignancy and two underwent changes in the chemotherapy regimen after PET-MRI revealed features of disease progression or relapse (Table 4). PET-MRI proved valuable in guiding treatment decisions by clarifying the tumour characteristics in an immunotherapy patient. In case 24, a patient with right frontal glioblastoma and Lynch syndrome experienced persistent disease after surgical resection and received regular immunotherapy. Although an interval MRI scan indicated tumour growth, it could not distinguish between residual tumour and pseudoprogression. However, PET-MRI revealed some FET uptake, suggesting active residual disease. Consequently, the decision was made to continue immunotherapy and monitor the lesion with a subsequent scan, thereby eliminating the need for a biopsy to assess the lesion’s nature.

**Figure 4:**
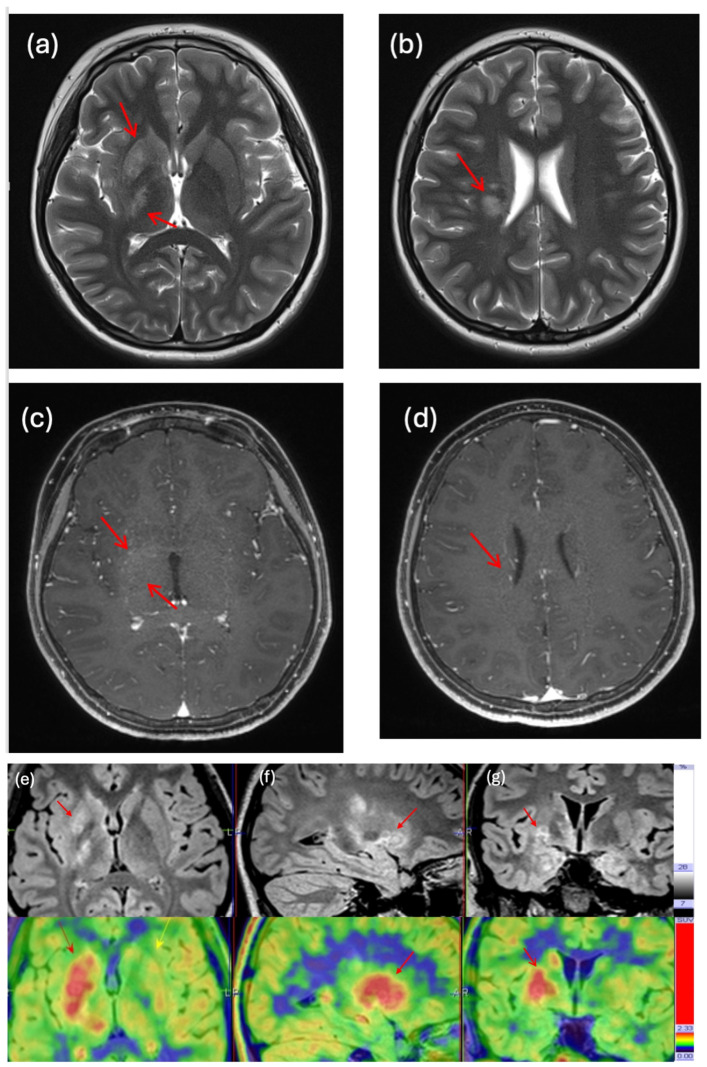
An 11-year-old boy (No. 3) presented with progressive left hemiparesis, initially diagnosed as ischemic stroke on the first MRI. PET-MRI helped to make the diagnosis of basal ganglia germinoma. (**a**,**b**) MRI showing hyperintense T2-weighted signal change at right basal ganglia extending to corona radiata with minimal mass effect on T2-weighted axial sequence (arrow). (**c**,**d**) MRI showing no significant enhancement along the whole-signal abnormality on post-gadolinium T1-weighted sequence (arrow). (**e**) Axial view, yellow arrow indicating normal contralateral side of the basal ganglia. (**f**) sagittal view, (**g**) coronal view of MET PET-MRI of the same patient showed a hyperintense T2-weighted/FLAIR signal at the right basal ganglia to corona radiata (upper row, red arrows). It demonstrated strong C11-methionine tracer uptake on PET-MRI (lower row, red arrows).

The majority of the PET-MRI scans helped supplement the diagnosis of CNS-GCT following a histological or image-guided diagnosis. In one case, there was diagnostic ambiguity, as the initial MRI identified the brain lesion as an infarct, while the PET-MRI demonstrated high uptake, suggesting a malignant germ cell tumour (No. 3, Table 3, Figure 4). In another case of bifocal germinoma, the initial diagnostic MRI only identified a pineal tumour but not a suprasellar lesion. Subsequent FET PET-MRI identified high metabolic uptake over suprasellar and pineal lesions, thereby confirming the diagnosis of a bifocal lesion (No. 14, Table 3). Two cases underwent PET-MRI after the completion of treatment to exclude relapse and were able to differentiate post-treatment changes from residual lesions (Nos. 9 and 10, Table 3). Their MRI findings identified remaining enhancing soft tissue areas that were inconclusive of residual lesions and recurrent disease, which the amino acid PET-MRI images revealed had low metabolic uptake, suggesting that significant active disease was unlikely. Hence, it was recommended to arrange surveillance scans without initiating active treatment.

**Figure 5:**
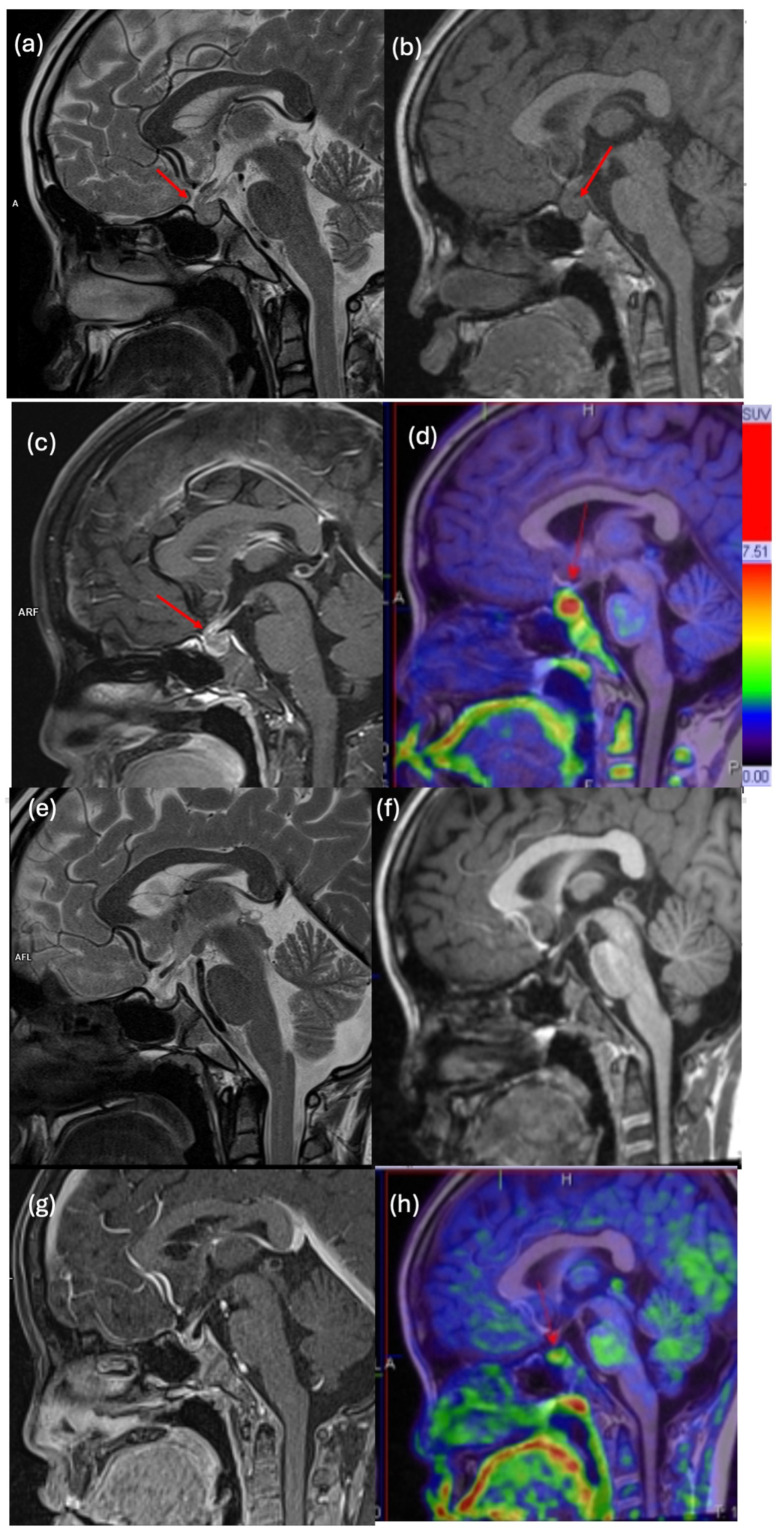
Suprasellar germinoma of a 14-year-old boy (No. 2). (**a**) T2-weighted sagittal scan showed pituitary stalk thickening with a prominent size of the pituitary gland. (**b**) Absent normal posterior pituitary T1-weighted bright spot on T1-weighted sagittal scan. (**c**) Enhancement on post-gadolinium T1-weighted scan and (**d**) strong C11-methionine tracer uptake noted within the pituitary gland and thickened pituitary stalk on PET-MRI [arrows]. (**e**) PET-MRI at 3 months post-treatment showed a significant interval decrease in size of the intrasellar mass with minimal residual pituitary stalk thickening on T2-weighted sagittal scan. (**f**) Absent normal posterior pituitary T1-weighted bright spot on T1-weighted sagittal scan. (**g**) Post-gadolinium T1-weighted scan. (**h**) Normalization of the MET tracer uptake in the pituitary gland without discernible MET uptake along the pituitary stalk on C11-methionine PET-MRI, indicating complete metabolic response [arrows]. There was no significant MET tracer uptake on further surveillance MET PET-MRI.

**Figure 6:**
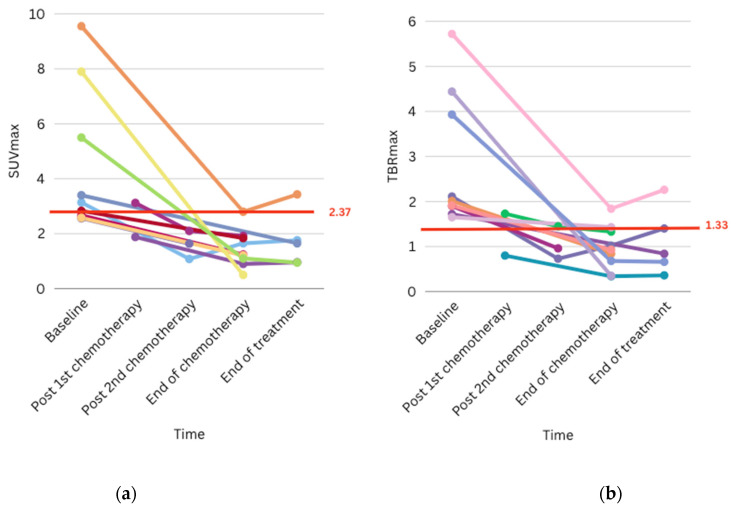
Graph showing the trend of SUVmax (**a**) and TBRmax (**b**) of eleven patients during treatment. Each color line represents a patient.

PET-MRI has also demonstrated an important role in monitoring treatment response. Eleven patients underwent serial PET-MRI scans to monitor treatment response, with five achieving complete metabolic responses following treatment completion (Figure 5) and six achieving favourable metabolic responses after chemotherapy completion. Notably, for early response assessment, two germinoma patients exhibited favourable and complete metabolic responses following two cycles of chemotherapy, respectively (Nos. 6 and 14, Table 2), while another achieved an excellent metabolic response after one cycle of chemotherapy only (No. 7, Table 2). Figure 6 plots the values of SUVmax and TBRmax of these patients against time during treatment; the majority of them had metabolic activities drop below the cut-off that distinguishes high-grade from low-grade lesions after completion of chemotherapy (Figure 6).

## 4. Discussion

Recent years have witnessed a paradigm shift in the application of metabolic imaging for CNS neoplasms. Amino acid PET demonstrates a significantly lower background uptake in normal brain tissue, thereby offering superior contrast resolution for the imaging of brain tumours compared to FDG-PET [32]. Compared to FDG, amino acid tracers are also less susceptible to interference from inflammation, making them an optimal choice for metabolic imaging of CNS neoplasms. Despite the well-known application of amino acid PET in adult brain tumours, its application in paediatric CNS neoplasms remains under investigation, primarily due to the heterogeneous nature of these diseases. The most extensive prospective study, conducted by Marner et al., demonstrated the advantageous role of incorporating FET PET alongside MRI in enhancing specificity and accuracy in differentiating paediatric CNS tumours from non-tumour lesions [22]. Nonetheless, more studies have begun to investigate the role of amino acid PET-CT in paediatric CNS neoplasms, with few focusing on amino acid PET-MRI. To our knowledge, one prior PET-MRI study on paediatric CNS neoplasms has been reported, but it focused exclusively on postoperative lesions [24]. In contrast, our cohort represents the largest PET-MRI analysis to date (n = 37), encompassing diverse tumour types and treatment-naïve lesions. Furthermore, we provide novel quantitative thresholds derived from PET-MRI parameters, offering an objective framework to distinguish neoplastic from non-neoplastic lesions.

Here, we report a local retrospective study of the clinical utility of amino acid PET-MRI in paediatric CNS neoplasms with direct comparison between high-grade lesions and low-grade or non-oncological lesions. Our cohort of 37 paediatric patients underwent 63 amino acid PET-MRI scans, demonstrating that PET-MRI effectively differentiates high-grade CNS neoplasms from low-grade lesions or non-oncological conditions. Our cohort identified a TBRmax cut-off of 1.62 to differentiate high-grade CNS lesions from low-grade/non-oncological lesions. Previous studies reported TBRmax thresholds of 1.3–1.5 for MET PET and 2.5 for FET PET in adult gliomas [3]. While other studies have noted a higher threshold for FET PET compared to MET PET, our analysis found no significant differences in SUVmax and TBRmax between the two groups in pre-treatment scans.

This study is the first to validate the use of amino acid PET-MRI in paediatric populations for this entity, achieving high diagnostic sensitivity and specificity through these metrics. Some studies demonstrate the efficacy of MET PET in distinguishing between tumorous and non-tumorous brain lesions; however, they are unable to differentiate between high-grade and low-grade brain lesions based on MET uptake [16,34]. Moreover, MET is less favoured than FET nowadays due to its shorter half-life, which poses technical difficulties in meeting the requirement for onsite cyclotron production. The clinical utility of amino acid PET-MRI was also assessed in our cohort, indicating that half of the patients benefited from this imaging modality in terms of diagnosis and tumour grading. Notably, in two-thirds of the patients, the integration of amino acid PET-MRI influenced clinical management by preventing unnecessary surgical interventions (such as biopsies or resections), refining diagnostic accuracy, or altering treatment strategies. Our findings corroborate those of Kertels et al., who demonstrated the beneficial impact of FET PET in guiding patient clinical management in 14 out of 21 patients with CNS tumours [13].

We also specifically investigated the role of amino acid PET-MRI in CNS-GCT, the second most common paediatric CNS tumour in East Asians, where MRI findings may be elusive. Several case reports have documented the utility of MET PET for the early detection and diagnosis of basal ganglia germinoma, localisation of biopsy targets, assessment of treatment response, and monitoring for residual tumour or relapse [29,31,32,36]. Two retrospective analyses have examined the role of MET PET and FDG PET in paediatric cohorts with intracranial germinoma [28,30]. We demonstrated the role of PET-MRI in differentiating CNS-GCT from other benign or low-grade lesions in the pituitary/suprasellar region and basal ganglia with high sensitivity and specificity. Among the eleven cases with pre-treatment imaging, metabolic activities were significantly higher compared to those of low-grade lesions or non-oncological diseases. Our findings align with those from Park YJ et al., who highlighted the significant value of SUVmax in distinguishing intracranial germinomas from non-CNS-GCTs. However, no direct calculation of TBRmax was used in their study [28].

Additionally, our data underscore the emerging potential of amino acid PET-MRI in assessing therapeutic response in CNS-GCT, aligning with prior case reports [31,32]. While this investigation constitutes the largest cohort of CNS-GCT patients evaluated with serial PET-MRI for longitudinal treatment monitoring, its interpretative scope is constrained by incomplete long-term outcome metrics. Notably, our preliminary findings posit amino acid PET-MRI as a viable tool for early treatment response assessment. This hypothesis is mechanistically supported by prior evidence linking amino acid tracer avidity to proliferative activity, including Ki-67 indices and cell nuclear antigen expression in CNS malignancies [37,38]. Although limited by sample size, observed correlations between SUVmax and TBRmax with CSF HCG levels further reinforce its potential utility in disease monitoring. While the results appear promising, confirmation of our results’ reproducibility and clinical validity necessitates prospective trials with extended follow-up durations, comparative analyses with conventional MRI, and histopathological correlation.

Our study is limited by its retrospective nature and sample size, which reflect the restricted availability of amino acid PET-MRI in a single-centred facility and the rarity of paediatric CNS neoplasms. Not all patients in our cohort received upfront pre-treatment amino acid PET-MRI due to the constraint of clinical urgency for treatment. Also, referrals for PET-MRI were determined on a case-by-case basis by multidisciplinary teams, since PET-MRI is not a routine diagnostic imaging modality in paediatric CNS neoplasms. This introduces potential selection bias. While the single-centre provenance of imaging data reduces interpersonal variability in scan interpretation, it may concurrently limit the generalizability of our findings to broader populations. Furthermore, our cohort exhibited a higher prevalence of CNS-GCTs compared to other CNS neoplasms. This observation is attributable to the diagnostic challenges inherent to CNS-GCTs, specifically germinomas, the predominant subtype in our cohort, which frequently present without elevated tumour markers and demonstrate nonspecific MRI features. In contrast, non-CNS-GCTs are often reliably identified through conventional MRI and typically necessitate urgent clinical intervention. The heterogeneity of our cohort, inherent to the disease spectrum and diagnostic practices, complicates inter-entity comparisons but underscores real-world challenges in paediatric neuro-oncology.

Moreover, some CNS neoplasms, such as germinomas, are characterized by a high degree of inflammation. It has been observed that MET PET can detect germinomas more effectively than non-germinomatous germ cell tumours, which may be partly due to its role in protein synthesis and higher uptake in inflammatory cells [10]. However, inflammation influences SUVs on amino acid PET and may result in false-positive values, and the correlation between the extent of intratumoral inflammation and metrics such as SUVmax or TBRmax remains to be elucidated. Notably, PET-MRI’s sensitivity to inflammatory process introduces diagnostic ambiguity, as evidenced in our cohort with a relatively high SUVmax and TBRmax seen in demyelinating disorder. The specificity of PET-MRI in differentiating neoplasms from inflammatory aetiologies should be depicted in prospective studies.

Nevertheless, our study provides preliminary insights into the feasibility of amino acid PET-MRI in management of paediatric CNS neoplasms, serving as a foundational framework for future prospective trials. Such studies should prioritize multicentre collaboration, standardized imaging protocols, and longitudinal outcome tracking to validate PET-MRI’s integration into standardized surveillance paradigms. Algorithmic integration of amino acid PET-MRI could enhance diagnostic workflows, particularly in cases where conventional MRI and biomarkers yield equivocal results. To optimize clinical applicability, future diagnostic algorithms should incorporate PET-MRI at critical decision points, such as inconclusive MRI findings before invasive procedures, ambiguous progression on MRI, or early response assessment. However, the clinical translation of amino acid PET-MRI in resource-limited settings remains constrained by cost and infrastructural barriers. Prioritizing cost-effective hybrid imaging strategies, telemedicine collaborations for centralized expertise, and selective deployment in diagnostically ambiguous cases could mitigate disparities in global neuro-oncology care.

## 5. Conclusions

In summary, our cohort provides robust pilot data on the utility of amino acid PET-MRI in paediatric CNS neoplasms, including in CNS-GCTs. The modality demonstrated value in diagnosis, disease monitoring, and evaluation of suspected relapse or residual lesions. Notably, PET-MRI informed clinical decision-making in 65% of cases by supplementing conventional imaging to avoid unnecessary invasive procedures (e.g., biopsy/surgery), refine tumour grading, and tailor therapeutic strategies. Quantitative analysis revealed a significant association between tracer uptake intensity and malignancy grade, with optimal cut-off values established to distinguish high- from low-grade lesions. Additionally, preliminary data highlight PET-MRI’s potential role in early treatment response assessment for CNS-GCTs. Future multicentre studies are warranted to validate PET-MRI as an early response assessment tool for stratifying patients with CNS-GCT patients and optimizing risk-adapted therapies.

## Figures and Tables

**Table 1:** Literature review on recent publications on amino acid PET in paediatric CNS neoplasms.

Paper	Modality of PET	Tracer	Patient Number (Age)	Diagnosis	Indication of Scan	Conclusion
Pirotte et al., 2007 [19]	PET-CT	FDG, MET	126 patients	Brain tumour	Provide additional information as MRI was inconclusive; select biopsy target; delineate tissue for resection	PET images helped with surgical management at diagnostic, surgical, and postoperative steps
Lee et al., 2008 [31]	PET-CT	MET	3 (9, 10, and 14 years)	Germinoma (basal ganglia)	Facilitate diagnosis and monitor treatment response	MET PET images helped with diagnosis and disease evaluation
Kawai et al., 2009 [32]	PET-CT	MET	3 (9, 17, and 18 years)	Germinoma (basal ganglia, thalamus)	Monitor for treatment response	MET PET helped with treatment response monitoring and identified lesions not visualized on MRI scan
Galldiks et al., 2010 [16]	PET-CT	MET	39 (mean 15 ± 5 years)	Pilocytic astrocytoma, astrocytoma, glioblastoma, medulloblastoma, ependymoma, atypical teratoid rhabdoid tumour, desmoplastic infantile ganglioglioma, non-tumour lesions, etc.	Differentiate tumours and non-tumourous brain lesions	MET PET images can differentiate tumourous and non-tumourous brain lesions at a threshold of 1.48 but are unable to differentiate between high-grade and low-grade tumours
Pirotte et al., 2010 [12]	PET-CT	FDG, MET	85 patients	Glioblastoma, anaplastic astrocytoma, ganglioglioma, oligodendroglioma, pilocytic astrocytoma, primitive neuroectodermal tumour, ependymoma, CNS-GCT, etc.	Plan for biopsy target when MRI unable to yield accurate information	PET-CT data influenced surgical decisions and procedures by differentiating indolent and active components, improving target selection and diagnostic yield of biopsies, providing prognostic information, reducing tissue amount needed for biopsy, delineating lesions better, guiding resection, improving detection of residual tumour, and avoiding unnecessary reoperation
Okochi et al., 2013 [30]	PET-CT	FDG, MET	10 patients (mean 13.4 ± 7 years)	CNS-GCT	Diagnosis	MET is a good tracer for diagnosing CNS germinoma and is useful for planning biopsies; FDG on the other hand is unable to produce sufficient image contrast
Dunkl et al., 2014 [17]	PET-CT	FET	48 patients (median 13 years)	Brain tumour	Diagnosis, treatment response, residual evaluation	FET PET helped with diagnosis, monitoring treatment response, and detecting residuals
Misch et al., 2015 [33]	PET-CT	FET	26 patients (median 12 ± 6.6 years)	Astrocytoma, ependymoma, pilocytic astrocytoma, glioblastoma, ganglioglioma, medulloblastoma, dysembryoplastic neuroepithelial tumour, etc.	Surgical planning	FET PET helped with target selection with decent specificity and high sensitivity
Lucas Jr. et al., 2017 [26]	PET-CT	MET	31 patients (median 10.5 years)	High-grade glioma	Prognosis	MET PET delineates regions at increased risk of recurrence and improves prognostic assessment and target definition for radiotherapy
Marner et al., 2019 [24]	PET-MRI	FET	22 patients (mean 9.5 years)	Diffuse midline glioma, atypical teratoid rhabdoid tumour, CNS-GCT, ganglioglioma, neuroepithelial tumour, ependymoma, pilocytic astrocytoma, etc.	Postoperative assessment	FET PET-MRI, when supplemented with MRI, improves specificity for detecting residual tumours
Grosse et al., 2021 [34]	PET-CT	FET	17 patients (median 12 years)	Medulloblastoma, low-grade glioma, high-grade glioma, CNS-GCT, choroid plexus tumour	Treatment response assessment	FET PET discriminated between residual/recurrent tumours and post-therapeutic changes
Park et al., 2022 [28]	PET-CT	MET	21 patients (median 16 years)	CNS-GCT	Diagnosis and monitoring	MET PET is useful as a diagnostic tool for CNS-GCT; PET avidity also strongly correlates with pre-treatment serum HCG
Kertels et al., 2023 [13]	PET-CT	FET	21 patients (mean 8.6 ± 5.2 years)	Glioblastoma, low-grade glioma, diffuse intrinsic pontine glioma, CNS-GCT, optic pathway glioma, primitive neuroectodermal tumour, pilocytic astrocytoma, ependymoma, etc.	Diagnosis confirmation, treatment planning	FET PET impacted patient management by avoidance of invasive surgery or biopsy, biopsy guidance, change in treatment, and confirmation of diagnosis
Rosen et al., 2025 [35]	PET-CT and PET-MRI	FET	80 patients from studies by Marner et al. and Dunkl et al.	CNS neoplasms	Diagnosis of treatment-related changes (cost-effective analysis)	FET PET is cost-effective for identification of treatment-related changes in pre-treated CNS tumours in children

**Table 2:** Patient demographics and characteristics.

	n = 37
**Age (median, range, years)**	11.2 (0.6–17)
**Sex (n, percentage)**	
F	15 (41%)
M	22 (59%)
**Grading (n, percentage)**	
High-grade	21 (56%)
Low-grade	8 (22%)
Non-oncological	8 (22%)
**Diagnosis (n, percentage)**	
CNS-GCT	15 (40%)
Thickened pituitary stalk (non-oncological)	7 (19%)
Low-grade glioma	6 (16%)
High-grade glioma	4 (11%)
Medulloblastoma	2 (5.5%)
Langerhans cell histiocytosis	2 (5.5%)
Demyelination	1 (3%)
**Site**	
Pituitary/suprasellar	18 (49%)
Pineal gland	6 (16%)
Cerebellum	5 (14%)
Basal ganglia	4 (11%)
Brainstem	3 (8%)
Ventricles	3 (8%)
Frontal lobe	2 (5%)
Cerebellopontine angle	1 (3%)
Spine	1 (3%)
**Tracer (N = no of scans, percentage)**	**N = 63**
FET	48 (76%)
MET	15 (24%)
**Indication of scan (N = no of scans, percentage)**	**N = 63**
Diagnosis	26 (41%)
Monitor/response assessment	30 (48%)
Evaluation of residual lesion or suspected relapse	7 (11%)

Abbreviations: CNS-GCT, central nervous system germ cell tumours; FET, 18-fluoroethyl-L-tyrosine; MET, C-11 methionine; n, number of patients; N, number of scans.

**Table 3:** CNS-GCT patient characteristics and first PET-MRI findings.

No	Sex	Age	Diagnosis	Stage	CSF AFP	CSF HCG	Serum AFP	Serum HCG	Treatment	Outcome	Purpose	SUVmax	TBRmax	Follow Up (Months)
1	M	17	Bifocal germinoma (Pineal, suprasellar, multiventricular)	M	<3	66	<3	1	4 cycles VP/C + WVI + PB	Relapse	Dx	5.5	3.93	26
2	M	14	Suprasellar germinoma	M0	<3	<1	<3	<2	4 cycles VP/C + WVI + PB	CR	Dx	9.55	5.72	29
3	M	11	Right basal ganglia germinoma	M0	<2	<1	<3	<1	4 cycles VP/C + WVI + PB	CR	Dx	3	1.78	63
4	M	13	Basal ganglia germinoma	M0	3	30	3	12	4 cycles VP/C + WVI + PB	CR	Dx	2.58	1.65	34
5	F	9	Bifocal germinoma (suprasellar, 4th ventricle)	M	3	124	3	377	6 cycles C/VP/Ifo + CSI + WVI + PB	CR	Dx	7.9	4.44	23
6	M	16	Suprasellar germinoma	M0	<3	1	<3	<1	4 cycles VP/C + WVI + PB	CR	Dx	3.14	2.11	8
7	F	17	Suprasellar germinoma	M0	<1	33	1	3	4 cycles VP/C + WVI + PB	CR	Assess response (post 1st chemo)	1.88	0.8	7
8	F	10	Suprasellar germinoma	M0	3	148	3	14	6 cycles of C/VP/Ifo + WVI + PB	CR	Dx	3.4	1.72	21
9	M	14	Suprasellar germinoma	M0	3	7	3	2.1	4 cycles VP/C+ WVI + PB	CR	Rule out relapse	2.3	1.53	22
10	M	8	NGGCT (left basal ganglia, ventricle)	M	26	35	18	24	6 cycles of C/VP/Ifo + WVI + PB	CR	EOC	1.05	1.18	19
11	M	16	Germinoma (pineal)	M0	<3	8	<3	1	4 cycles VP/C	NA	Assess response (post 1st chemo)	3.12	1.73	3
12	M	12	Bifocal germinoma (suprasellar, pineal)	M0	<3	24	<2.5	13	4 cycles VP/C	NA	Dx	2.67	2.01	3
13	M	15	Bifocal germinoma (suprasellar, pineal)	M0	<3	59	3	12	4 cycles VP/C	NA	Dx	2.83	1.91	3
14	M	11	Bifocal germinoma (suprasellar, pineal)	M0	<3	7	1	<1	4 cycles VP/C	NA	Dx	2.56	1.9	2
15	M	14	Germinoma (pineal)	M0	<3	4	<3	2	4 cycles VP/C	NA	Dx	5	2.12	1

Unit for age: years. Units for CSF and serum HCG: IU/L; CSF and serum AFP: ng/mL. Abbreviations: AFP, alpha fetoprotein; C, carboplatin; CR, complete remission; CSI, craniospinal irradiation; CSF, cerebrospinal fluid; Dx, diagnosis; EOC, end of chemotherapy; HCG, beta human chorionic gonadotrophin; Ifo, ifosfamide; M, metastatic disease; M0, no metastatic disease; NA, not available; NGGCT, non-germinomatous germ cell tumour; PB, involved field boost; VP, etoposide; WVI, whole-ventricular irradiation.

**Table 4:** Case illustration of the clinical impact of amino acid PET-MRI.

Avoidance of Biopsy/Surgery									
Patient No	Age (Years)	Sex	Diagnosis	Site	Indication of Scan	Tracer	SUVmax	TBRmax	Conclusion
15	9	F	Thickened pituitary stalk	Pituitary	Diagnosis	FET	1.09	0.91	Suggestive of non-neoplastic lesion, biopsy not recommended. Followed-up for 14 months.
16	14	M	Thickened pituitary stalk	Pituitary	Diagnosis	FET	1.74	0.83	Suggestive of non-neoplastic lesion, biopsy not recommended. Followed-up for 24 months.
9	14	M	Germinoma	Pituitary	Rule out relapse	FET	2.3	1.53	Favours post-treatment residual, not indicated for biopsy/resection.
17	7	F	Thickened pituitary stalk	Pituitary	Diagnosis	FET	0.87	0.64	Suggestive of non-neoplastic lesion, biopsy not recommended. Followed-up for 21 months.
18	1	M	Congenital pontine tumour	Brainstem	End-of-treatment evaluation for residual tumour	MET	1.05	0.76	MRI after chemotherapy showed no reduction in tumour size. PET-MRI showed low metabolic activity, suggestive of post-treatment changes. Biopsy not recommended. CSF molecular study suggestive of low-grade lesion. Maintenance weekly vinblastine given till 1 year old. Patient followed-up for 2 years without progression.
19	7	M	LCH of frontal bone	Pituitary	Diagnosis/staging	FET	0.81	0.82	MRI showed suspected pituitary stalk thickening; PET-MRI showed no increase in metabolic uptake, not suggestive of LCH involvement of pituitary. Biopsy avoided. Spontaneous resolution and followed-up for 2 years.
20	4	M	Low-grade glioma	Basal Ganglia	Diagnosis	FET	2.18	1.01	Low metabolic uptake, suggestive of low-grade lesion; biopsy not recommended. Later developed seizure with biopsy confirming low-grade lesion.
21	10	F	Thickened pituitary stalk	Pituitary	Diagnosis	FET	1.6	0.75	Suggestive of non-neoplastic lesion, biopsy not recommended. Followed-up for 17 months.
22	8	F	Thickened pituitary stalk	Pituitary	Diagnosis	MET	2.6	1.93	Suggestive of non-neoplastic lesion, biopsy not recommended. Followed-up for 28 months.
23	17	F	Thickened pituitary stalk	Pituitary	Diagnosis	FET	1.42	0.85	Suggestive of non-neoplastic lesion, biopsy not recommended.
	19				Monitor	FET	1.76	0.89	Follow-up MRI showed interval increase in size of thickened stalk; FET PET showed no increased metabolic uptake; plan for continued surveillance. Followed-up for 23 months.
24	15	F	GBM	Frontal lobe	Evaluate residual	FET	3.4	2.01	MRI unable to differentiate pseudoprogression versus true progression; PET-MRI showed increased FET uptake suggestive of possible residual active neoplasm; surveillance suggested. Decision-making facilitated with the additional information from PET-MRI without the need for biopsy.
25	7	F	Thickened pituitary stalk	Pituitary	Diagnosis	FET	1.31	0.79	Suggestive of non-neoplastic lesion, biopsy not recommended. Followed-up for 18 months.
26	6	M	Medulloblastoma	Cerebellum	Rule out relapse	FET	0	0	MRI showed new enhancing focus. No FET uptake suggestive of active high-grade lesion. Surgery or biopsy avoided. In remission for 21 months.
10	8	M	NGGCT	Basal ganglia	Evaluate residual	FET	0.73	0.82	MRI showed residual lesion; PET-MRI showed no increase in metabolic uptake, suggestive of post-treatment changes. In remission for 18 months.
**Refine tumour grading/diagnosis**									
27	4	M	Diffuse midline glioma	Brainstem	Diagnosis	FET	6.16	3.76	Biopsy result unable to differentiate low-grade or high-grade glioma due to low cellularity on tissue slides. PET-MRI showed intra-lesional metabolic heterogeneity and high metabolic uptake in the unbiopsied region. Next-generation sequencing later revealed H3F3A mutation.
28	16	M	GBM	Frontal	Diagnosis	MET	4	1.8	Surgical resection with pathology showed low-grade glioma but high MET uptake suggestive of high-grade lesion. Eventually methylation revealed diagnosis of glioblastoma.
29	2	M	Pilocytic astrocytoma	Cerebellum	Diagnosis	MET	1.99	1.34	MRI unable to differentiate high-grade or low-grade lesion; PET MRI showed mild increase in uptake suggestive of low-grade neoplasm. Eventually had surgical resection due to increased tumour size; diagnosis of pilocytic astrocytoma made.
3	11	M	Germinoma	Basal ganglia	Diagnosis	MET	3	1.78	First MRI suggestive of infarction; high MET uptake on PET-MRI suggestive of high-grade neoplasm.
14	11	M	Germinoma	Suprasellar and pineal	Diagnosis	FET	2.56	1.9	First MRI identified only the pineal lesion; PET-MRI showed high metabolic uptake over suprasellar region and pineal region; diagnosis of bifocal germinoma made after histological confirmation of suprasellar lesion.
**Treatment guidance or modification**									
30	17	F	LCH	CP angle	Evaluate residual	FET	1.39	0.55	Post-treatment MRI showed similar residual lesion compared to pre-treatment scan; unsure whether active lesion or not. PET-MRI showed mild increased FET uptake, possible remaining active lesions. Surgery performed for residual lesion.
31	1	F	Ganglioglioma	Cerebellum	Diagnosis	MET	1.53	1.25	MRI showed incidental enhancing lesion; PET-MRI showed mild MET uptake, suggestive of low-grade neoplasm; hence decision was made for surgical resection.
32	16	M	Pilocytic astrocytoma	Cerebellum	Diagnosis	FET	1.94	1.59	Initial MRI showed 2 cm cerebellar lesion; observation planned; repeated scan with PET-MRI showed high uptake suggestive of neoplasm; hence decision was made for surgical resection.
33	8	M	Medulloblastoma	Spine	Evaluate relapse	FET	1.82	NA	MRI spine inconclusive for isolated spinal relapse; high uptake on PET-MRI supported diagnosis of spinal metastasis; treatment for relapse medulloblastoma initiated with response.
34	0.75	F	High-grade glioma (ROS1 fusion positive)	Temporal-parietal	Response assessment	FET	2.73	1.16	MRI inconclusive for residual tumour; PET-MRI showed high uptake, suggestive of disease progression, hence switched to targeted therapy.

Abbreviations: CP angle: cerebellopontine angle; GBM: glioblastoma; NGGCT, non-germinomatous germ cell tumour; LCH: Langerhans cell histiocytosis. The cases are categorized by clinical utility of PET-MRI, namely avoidance of biopsy/surgery, refine tumour grading/diagnosis and treatment guidance or modification (Bold).

## Data Availability

The raw data supporting the conclusions of this article will be made available by the authors on request.

## Abbreviations

The following abbreviations are used in this manuscript:

AFP	Alpha fetoprotein
ASCT2	Alanine-serine-cysteine transporters
AUC	Area under the curve
AutoHSCT	Autologous haematopoietic stem cell transplantation
C	Carboplatin
CI	Confidence interval
CMR	Complete metabolic response
CNS	Central nervous system
CNS-GCT	Central nervous system germ cell tumour
CP angle	Cerebellopontine angle
CR	Complete remission
CSI	Craniospinal irradiation
CSF	Cerebrospinal fluid
DOPA	Fluorodopa
DTI	Diffusion tensor imaging
DWI	Diffusion-weighted imaging
Dx	Diagnosis
EOC	End of chemotherapy
EOT	End-of-treatment evaluation
FDG	Fluorodeoxyglucose
FET	18-fluoroethyl-L-tyrosine
FLAIR	Fluid-attenuated inversion recovery
GBM	Glioblastoma
HCG	Beta human chorionic gonadotrophin
Ifo	Ifosfamide
LAT1	L-type amino acid transporters
LCH	Langerhans cell histiocytosis
M	Metastatic disease
M0	No metastatic disease
MET	C-11 methionine
MRI	Magnetic resonance imaging
MRS	MR spectroscopy
n	Number of patients
N	Number of scans
NA	Not available
NGGCT	Non-germinomatous germ cell tumour
PET-CT	Positron emission tomography–computer tomography
PET-MRI	Positron emission tomography–magnetic resonance imaging
PB	Involved field boost
ROC	Receiver operating characteristic curve
ROI	Region of interest
SWI	Susceptibility-weighted imaging
SUVmax	Maximal standardized uptake values
TBRmax	Tumour-to-background SUVmax ratios
VOI	Volume of interest
VP	Etoposide
WVI	Whole-ventricular irradiation
